# Supplementary figures and images for: Capturing the differences between humoral immunity in the normal and tumor environments from repertoire-seq of B-cell receptors using supervised machine learning
Source: BMC Bioinformatics. 2019 May 28;20:267. doi: 10.1186/s12859-019-2853-y (PMC6537402; doi:10.1186/s12859-019-2853-y)

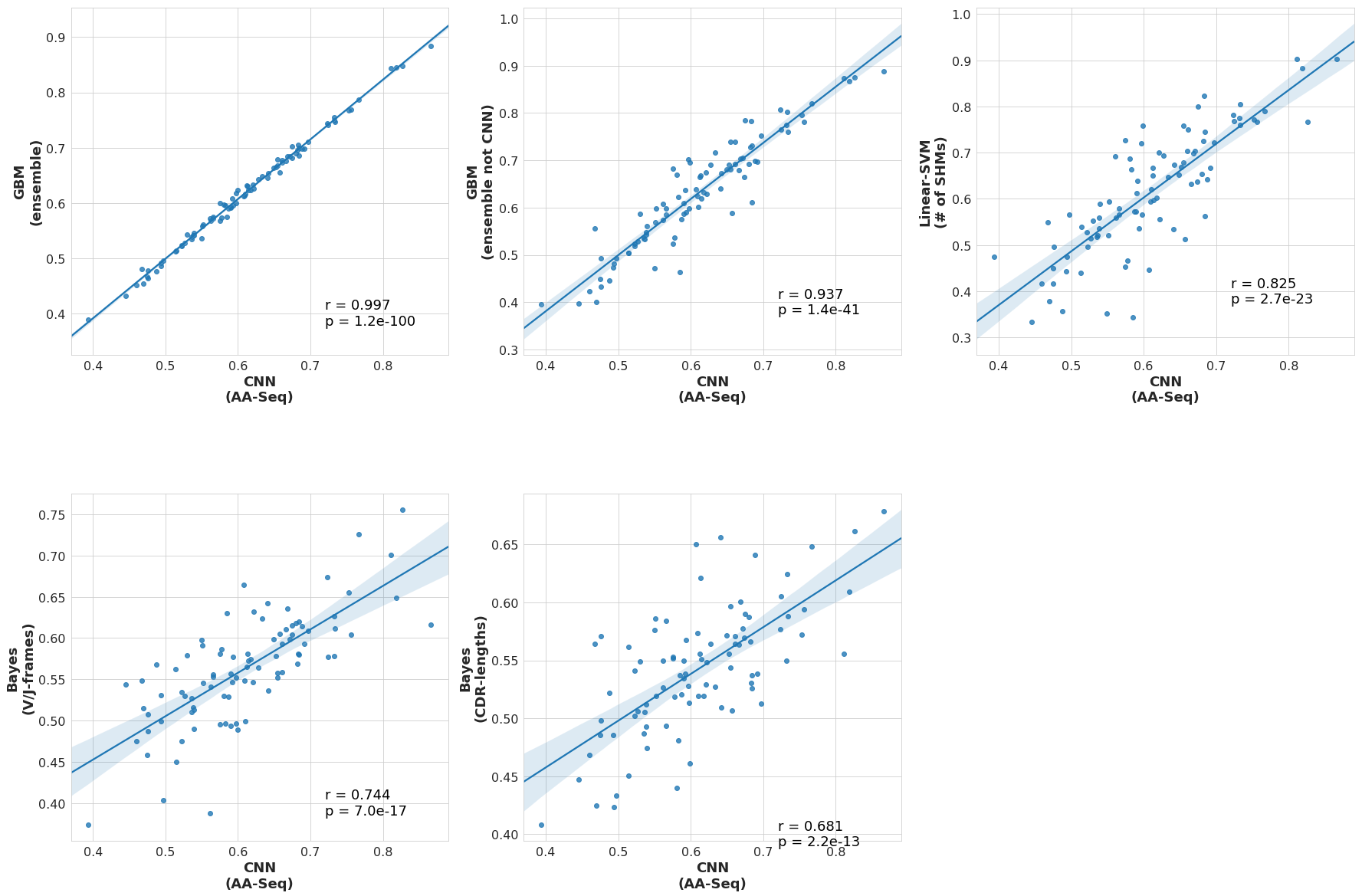

Supplement: Supplementary file 2 — Figure S1. Scatter plots showing correlation between 89 AU-ROCs of CNN to those of other classifiers (ensemble classifier, ensemble classifier without CNN, linear classifier using # of SHMs, Bayes classifier using usage of V/J frames, and Bayes classifier using CDR-lengths. (PNG 188 kb) [file 12859_2019_2853_MOESM2_ESM.png]

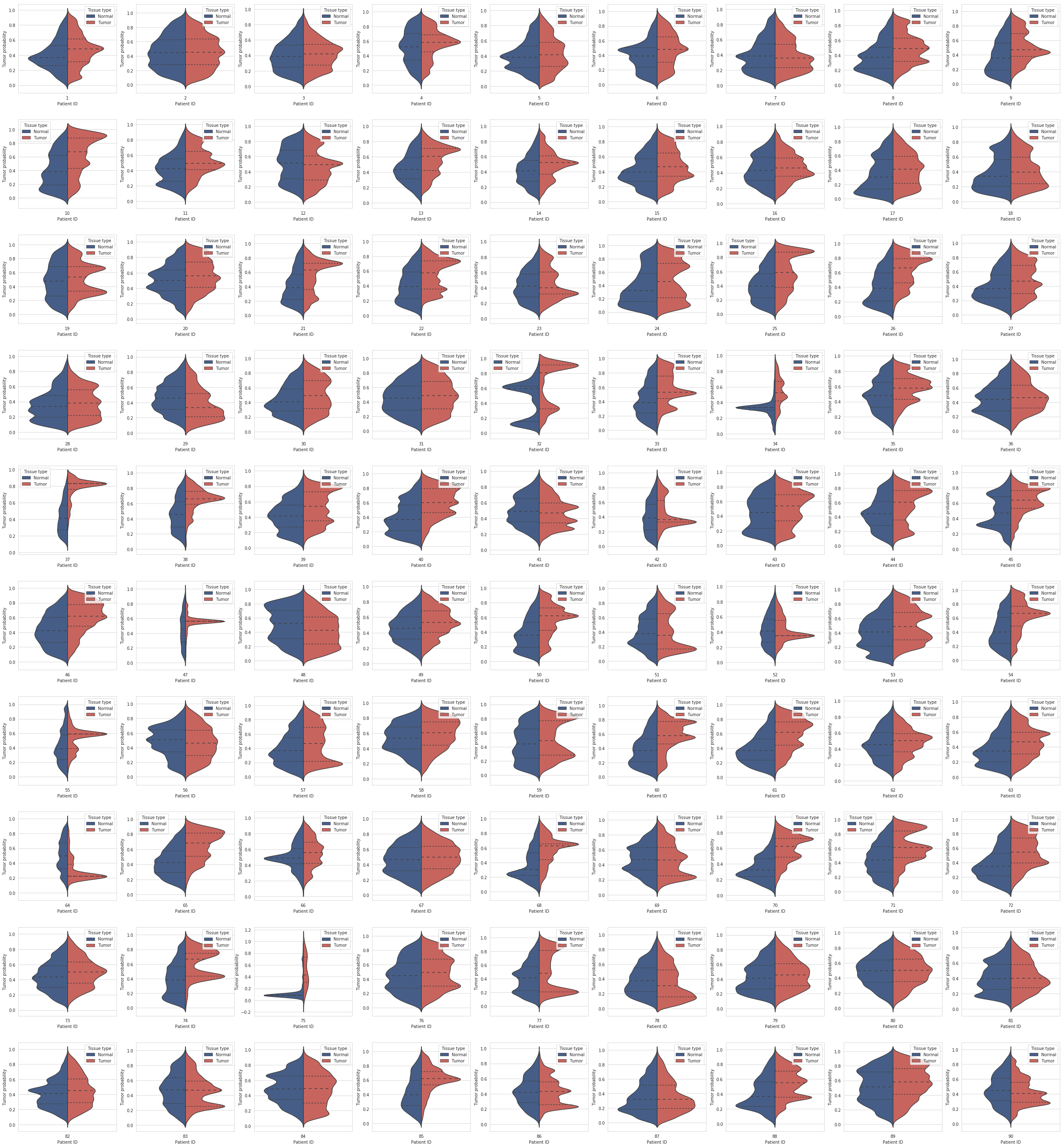

Supplement: Supplementary file 3 — Figure S2. Clonal entropy and distribution of tumor probabilities in whole normal/tumor samples over 90 patients. (PNG 889 kb) [file 12859_2019_2853_MOESM3_ESM.png]
